# Administration of recombinant human thrombopoietin is associated with alleviated thrombocytopenia in adult intensive care unit patients with pneumonia: A single-center retrospective study

**DOI:** 10.3389/fphar.2022.1007719

**Published:** 2022-10-10

**Authors:** Bailiang Chen, Jiabin Xuan, Feng Wu, Nengxian Shi, Jianwei Dai, Shumin Cai, Shengli An, Qiaobing Huang, Xiaoling Huang, Zhongqing Chen, Zhenhua Zeng

**Affiliations:** ^1^ Department of Critical Care Medicine, Nanfang Hospital, Southern Medical University, Guangzhou, China; ^2^ Department of Critical Care Medicine, The Second Affiliated Hospital of Shantou University Medical College, Shantou, China; ^3^ Department of Biostatistics, School of Public Health (Guangdong Provincial Key Laboratory of Tropical Disease Research), Southern Medical University, Guangzhou, Guangdong, China; ^4^ Guangdong Provincial Key Lab of Shock and Microcirculation, Department of Pathophysiology, Southern Medical University, Guangzhou, China; ^5^ Department of Pediatrics, The First Affiliated Hospital of Shantou University Medical College, Shantou, China

**Keywords:** recombinant human thrombopoietin (rhTPO), thrombocytopenia, pneumonia, platelet counts, hemorrhage

## Abstract

**Background:** Recombinant human thrombopoietin (rhTPO) is reported to stimulate platelet production and increase peripheral platelet counts; it is primarily used to manage chemotherapy-induced thrombocytopenia and idiopathic thrombocytopenic purpura. However, the effect of rhTPO in patients with pneumonia and thrombocytopenia remains uncertain.

**Objective:** To assess the association of rhTPO and platelet counts in ICU patients with pneumonia and thrombocytopenia.

**Materials and Methods:** A retrospective cohort study was performed in the ICU department, Nanfang Hospital, Southern Medical University, Guangzhou, China. From January 2016 to April 2021, patients with pneumonia and thrombocytopenia were allocated to two groups—the rhTPO and no-rhTPO groups—according to whether they received rhTPO treatment or not during their ICU stay. Demographical and clinical data were collected and analyzed using statistical software; *p* < 0.05 was considered statistically significant.

**Results:** Out of 327 patients, 149 were in the rhTPO group and 178 were in the no-rhTPO group. Within the first 7 days, platelet counts increased more for patients in the rhTPO group compared with those in the no-rhTPO group (99.21 ± 102.613 vs. 2.08 ± 43.877, *p* = 0.000). The clinical recovery rate of platelets increased within 7 days (65.8 vs. 18.5%, *p* = 0.000) and, after 7 days of enrollment, hemorrhagic scores decreased more apparently in the rhTPO group (2.81 ± 2.856 vs. 1.16 ± 2.123, *p* = 0.000). Further, bleeding events ceased in 66.7% of the patients in the rhTPO group compared with 37.3% of the patients in the no-rhTPO group (*p* = 0.000). Less red-blood-cells transfusions were needed in the rhTPO group (3.639 ± 4.630 vs. 5.818 ± 6.858, *p* = 0.009). Furthermore, through logistic regression, rhTPO administration was found to be an independent indicator that affected the platelet recovery rate within 7 days.

**Conclusion:** This study finds that rhTPO administration is associated with increased platelet counts, alleviated bleeding, and reduced blood transfusion. For patients with pneumonia and thrombocytopenia, rhTPO may be an effective therapeutic drug; however, more RCT trails are needed to confirm our observation.

## Introduction

Platelets play a pivotal role in normal hemostasis and thrombus formation mirroring small and anucleate cell fragments. The primary function of platelets is to patrol the vasculature and immediately seal vessel breaches immediately to limit blood loss (Deppermann and Kubes 2016). Recent studies have notarized that platelets have a variety of biological functions in inflammatory balance, immune response, and tissue repair and regeneration, especially in infections (Jenne and Kubes 2015; Cedervall et al., 2018; Page and Pretorius 2020).

Thrombocytopenia, which is common in intensive care unit (ICU) patients (Khurana and Deoke 2017), is defined as a low-than-normal number of platelets in the blood, ranging from 13 to 44% (Claushuis et al., 2016). Thrombocytopenia was defined as a platelet count of less than 150×10^9^/L, but the new guidelines adjusted the cut-off value to 100 ×10^9^/L based on prospective studies (Matzdorff et al., 2018). Thrombocytopenia has a considerable impact on the prognosis of critically ill patients (Hui et al., 2011); it could lead to increased bleeding events and is an independent prognostic factor for patients with severe infections (Steéphan et al., 1999; Sharma et al., 2007).

Platelets originate from megakaryocytes, which develop primarily in the bone marrow, where platelet production is presumed to mainly occur (Lefrançais and Looney, 2019). However, mounting evidence suggests that the lung is a site of platelet biogenesis and a reservoir for hematopoietic progenitors (Lefrançais et al., 2017). Further, lung diseases exhibit alterations in platelet numbers and functions, especially infectious lung diseases (Gomez-Casado et al., 2019). Thrombocytopenia develops in 25–58% of ICU patients with community-acquired pneumonia (CAP) (Brogly et al., 2007; Dunaiceva and Sabelnikovs 2015). Furthermore, previous studies have found that thrombocytopenia marks poor pneumonia outcome (Mirsaeidi et al., 2010; Gorelik et al., 2017). Thrombocytopenia is associated with higher hospital mortality, while its resolution is related to better clinical prognosis of ICU patients (Dunaiceva and Sabelnikovs 2015). The premise of treatment is to remove or control the inducement of thrombocytopenia as soon as possible. However, it is difficult to determine the cause of thrombocytopenia in ICU and it is often multifactorial (Oguzulgen et al., 2004). Thus, several studies have suggested that platelet transfusion may reverse the low platelet counts in patients (Van der Linden et al., 2012) and supportive transfusion remains standard of care in treatment of thrombocytopenia in ICU. However, because of its high cost, short self-life, transfusion-related immune and infectious complications, ineffective transfusion and platelet antibody production, platelet transfusion is usually disputed and restricted to critically ill patients with thrombocytopenia (Etchill et al., 2017). Further, shortages of platelets are experienced worldwide. Therefore, the extensive application of platelet transfusion in clinical practice is limited and the management of thrombocytopenia remains a challenge (Humbrecht et al., 2018).

In recent years, clinical studies have illustrated that recombinant human thrombopoietin (rhTPO) can significantly stimulate platelet production and increase peripheral platelet counts, which is primarily used to manage chemotherapy-induced thrombocytopenia and primary immume thrombocytopenia (ITP) (Altomare et al., 2012; Rodeghiero and Carli 2017). rhTPO is a cytokine that promotes the differentiation of bone marrow hematopoietic stem cells into megakaryocytes and stimulates the growth, differentiation, and maturation of megakaryocytes. It is a highly specific platelet stimulator (Kuter 2009). However, the effect of rhTPO in patients with pneumonia and thrombocytopenia remains uncertain. Our study explores the impact of rhTPO in patients with pneumonia and thrombocytopenia. This single-center retrospective cohort study aimed to assess the association of rhTPO and platelet counts in critically ill patients with pneumonia and thrombocytopenia.

## Methods

### Study design and Patient identification

This was a retrospective cohort study in the department of critical care medicine (ICU), Nanfang Hospital of Southern Medical University, Guangzhou, China. This large-scale comprehensive hospital engaged in medical treatment, medical research, and medical education. The patients were admitted to ICU between January 2016 and April 2021.

Pneumonia was diagnosed according to the following criteria: the presence of acute illness with ≥2 symptoms or signs of lower respiratory tract infection (cough or expectoration; hemoptysis; pleuritic chest pain; shortness of breath; temperature >37.3°C; and crackles or bronchial breath sounds) and a new chest radiographic infiltrate evidence (Eurich et al., 2015; Kalil et al., 2016; Metlay et al., 2019).

Inclusion criteria were as follows: 1) age ≥18 years; 2) patients admitted to the ICU; 3) diagnosis or clinical diagnosis of pneumonia; and 4) peripheral blood platelet counts <100×10^9^/L.

Patients’ exclusion criteria were as follows: 1) history of hematopoietic stem cell transplantation or liver, kidney, lung or other solid organ transplantation; 2) malignant hematologic disorders; 3) and immune thrombocytopenia, such as ITP, systemic lupus erythematosus, and thrombotic thrombocytopenic purpura. Other criteria included 4) chemotherapy or radiation within the last 2 weeks; (5) thrombocytopenia caused by gastrointestinal bleeding or infections in other sites; 6) hyperplenism in advanced cirrhosis; 7) being on immunosuppressive therapy; 8) end-stage renal disease; 9) length of ICU stay <3 days; and (10) pregnancy and lactation.

The included patients were allocated to either the rhTPO group or the no-rhTPO group based on whether rhTPO was administrated during treatment. All patients were treated in accordance with the latest guidelines for pneumonia (Kalil et al., 2016; Metlay et al., 2019), including the use of sensitive antibiotics and organ support. Infusion of blood products, such as red blood cells and plasma, were given according to their corresponding indications. Platelet infusion was performed when platelet counts were <10×10^9^/L without apparent bleeding and when counts were <20 × 10^9^/L if the patient had a significant risk of bleeding. Higher platelet counts (50 × 10^9^/L) were advised for active bleeding, surgery, or invasive procedures (Estcourt et al., 2017; Rhodes et al., 2017). The rhTPO group was given a subcutaneous injection of 15000U rhTPO per day (Wu et al., 2014). The no-rhTPO group received the same treatment as the rhTPO group except rhTPO administration.

### Data collection

The following data were obtained from the patients’ electronic medical records: gender, age, patients’ type (medical/surgical), underlying diseases and comorbidities. Thereafter, the clinical characteristic baseline and laboratory results were collected during the first 24 h after enrollment, including markers of organ functions (heart, liver, renal and coagulation functions) and biomarkers of infection (procalcitonin and C-reactive protein). Severity of illness scores for each patient record was calculated using the Acute Physiology and Chronic Health Evaluation II (APACHE II) classification system (Knaus et al., 1985), Sequential Organ Failure Assessment (SOFA) (Vincent et al., 1996), and the pneumonia severity index (PSI) (Fine et al., 1997) within 24 h after enrollment. The changes in platelet counts during the first week were recorded. Moreover, the markers of organ functions on the 7th day after enrollment were recorded as well. The severity of bleeding manifestations was assessed at inclusion and during the 7 days, using a previously reported standardized clinical scoring system, then the total hemorrhagic score was calculated by adding the scores for each item (Godeau et al., 2002). The higher the hemorrhagic score was, the more serious the bleeding. Patients’ blood transfusion volumes were counted for 28 days. If several pieces of measurements or data were collected on the same day, the worst or lowest data were retained.

### Definitions

In this study, thrombocytopenia was defined as platelet counts <100×10^9^/L, based on the hospital laboratory’s reference range. If more than one platelet counts records were available within 24 h, the lowest one was recorded. Clinical recovery of platelets was defined as platelet counts ≥100×10^9^/L or an increase in platelet counts ≥50 × 10^9^/L for at least 3 days. The clinical recovery time of platelets was calculated from the enrollment date to the date when clinical recovery was reached (Zhou et al., 2020). Platelet recovery rate within 7 days refers to the percentage of patients that experience clinical recovery of platelets within 7 days.

### Statistical analysis

Quantitative variables were reported using means ± standard deviation (SD) when they were distributed normally; in case of abnormal distributions, medians and 25th and 75th percentiles were used. Qualitative variables were presented as absolute numbers (percentages). Normality was tested using the Shapiro-Wilk test. Normally distributed data were compared through t-test or t' test (Satterthwaite approximate t-test, when equal variances were not assumed), whereas the Mann-Whitney *U* test was used to analyze abnormally distributed data. The enumeration data compared using Pearson Chi-Square or the Fisher exact method. The changes of laboratory indexes before and after treatment were analyzed through covariance analysis, in which the baseline data were treated as covariates. The relationships between platelet recovery rate within 7 days and the study variables were evaluated using a logistic regression model. *p* values less than 0.05 were considered statistically significant. Statistical analyses were performed using SPSS (Version 21.0. IBM, NY).

From the univariable analysis, combined with clinical expertise, we identified 11 variables for inclusion in the multivariable regression analysis of platelet recovery rate. Independent variables such as age, platelet counts, and disease severity scores were recoded, and regression equations were introduced. The specific methods of recoding are listed in Supplementary Table 1.

### Ethics approval and consent to participate

This study was performed in accordance with the Declaration of Helsinki and approved by the medical ethics committee of Nanfang Hospital of Southern Medical University (No. NFEC-202111-K24-01). Because the data were anonymous and observational, informed consent was waived.

## Results

### Demographics and baseline characteristics of Patients

**FIGURE 1:**
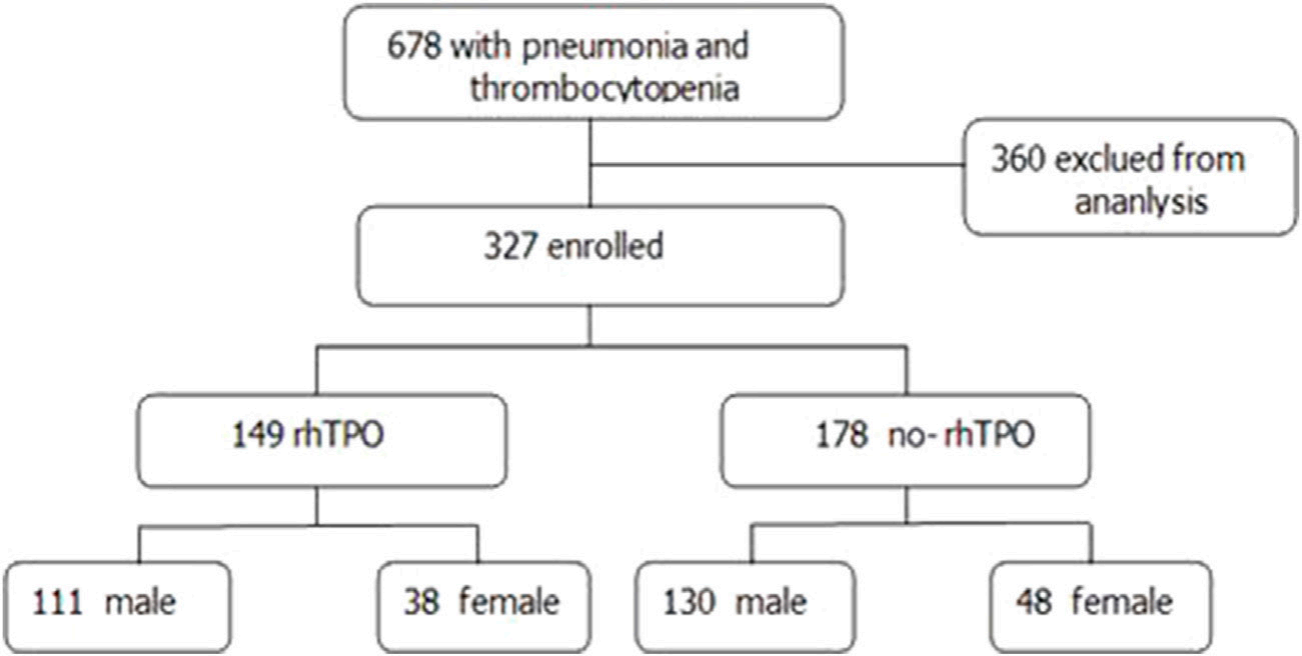
Flowchart.

Between January 2016 and April 2021, 687 ICU patients with pneumonia and thrombocytopenia in Nanfang Hospital, Southern Medical University, were enrolled. In total, 360 patients were excluded according to the inclusion and exclusion criteria. Finally, 327 patients with pneumonia, 241 male and 86 female, were included in the study (Figure 1).

The demographics and baseline characteristics of the patients are shown in Table 1. The rhTPO group had 149 cases, including 111 male and 38 female, with a mean age of 63.85 ± 16.766 years. The no-rhTPO group had 178 individuals, including 130 male and 48 female, with a mean age of 62.76 ± 17.032 years. No significant differences were found in terms of gender and age, antibiotic therapy, as well as history of hypertension, diabetes, coronary atherosclerotic heart disease, chronic heart failure, stroke, COPD, and chronic kidney disease between the rhTPO group and no-rhTPO groups (Table 1 and Supplementary Table S2).

**TABLE 1:** Demographics and baseline characteristics of patients.

Characteristics	rhTPO Group	no-rhTPO Group	*p* Value
Number, n	149	178	—
Male, n (%)	111, 74.5%	130, 73.0%	0.765
Age (years), median (IQR)	67, (54.5-76.5)	65.5, (51-77)	0.490
Comorbidities, n (%)			
Hypertension	59, 39.6%	59, 33.1%	0.226
Diabetes mellitus	44, 29.5%	42, 23.6%	0.225
Coronary atherosclerotic heart disease	16, 10.7%	18, 10.1%	0.853
Chronic heart failure	5, 3.4%	7, 3.9%	0.782
Stroke	15, 10.1%	15, 8.4%	0.609
COPD	16, 10.7%	19, 10.7%	0.985
Chronic kidney disease	14, 9.4%	22, 12.4%	0.394
Severity of disease			
APACHE II, median (IQR)	24, (20-28)	22, (18-25)	0.000
SOFA, median (IQR)	12, (10-14)	12, (9-13)	0.038
PSI, mean ± SD	151.06 ± 30.675	143.54 ± 31.192	0.030
Hemorrhage score (Godeau et al., 2002), mean ± SD	5.19 ± 4.311	4.61 ± 4.268	0.228
Hemorrhage events, n (%)	90, 60.4%	102, 57.3%	0.571
Gastrointestinal bleeding, n (%)	35, 23.5%	51, 28.7%	0.291
Cutaneous and mucosal bleeding, n (%)	28, 18.8%	36, 20.2%	0.745
Respiratory tract bleeding, n (%)	10, 6.7%	13, 7.3%	0.835
Urinary bleeding, n (%)	6, 4.0%	10, 5.6%	0.506

COPD; chronic obstructive Pulmonary disease; APACHE II; Acute Physiology and Chronic Health Evaluation II.

SOFA; sequential organ failure assessment; PSI; the Pneumonia Severity Index.

SD: standard deviation; IQR: 25th and 75th percentiles.

*p* value was generated using t-test, t' test, the Mann-Whitney *U* test or Pearson Chi-Square.

Compared to the no-rhTPO group, patients in the rhTPO group were more critical on ICU admission, as reflected by the higher APACHE II score (*p* = 0.000), SOFA score (*p* = 0.038) and PSI score (*p* = 0.030). Besides, the hemorrhage scores and events were between the two groups did not differ on admission (all *p* > 0.050), and the specific details are reflected in Table 1.

### Effects of rhTPO administration on Platelet counts

On the ICU admission day, platelet counts in the rhTPO group was lower than that in the no-rhTPO group (49.43 ± 23.291 vs. 73.01 ± 22.111, *p* = 0.000). However, on the third, fifth, and seventh day, the mean platelet counts in the rhTPO group were higher than that in the no-rhTPO group at the same timepoint, and the differences were all significant (all *p* < 0.01, Table 2). In addition, the minimum platelet counts within the 14days in the rhTPO group was seemed lower than that in no-rhTPO group (34.82 ± 19.029 vs. 37.54 ± 21.513) during treatment, but the difference was not statistically significant (*p* = 0.225). However, the maximum platelet counts (×10^9^/L) for the rhTPO group were significantly higher than that in the no-rhTPO group (256.89 ± 137.789 vs. 125.77 ± 74.076; *p* = 0.000; Table 2). Furthermore, the increases of platelet count in the first 7 days in the rhTPO group were significantly higher, compared with those in the no-rhTPO group (99.21 ± 102.613 vs. 2.08 ± 43.877, *p* = 0.000). Moreover, 65.8% of the patients in the rhTPO group reached the clinical recovery of the platelet counts within 7 days, compared to 18.5% in the no-rhTPO group, and the difference was statistically significant (*p* = 0.000).

**TABLE 2:** Platelet counts and therapeutic effects.

Variables	rhTPO Group	no-rhTPO Group	*p* Value
Changes of Platelet Counts			
Pre-treatment platelet counts, ×10^9^/L	49.43 ± 23.291	73.01 ± 22.111	0.000
The third day platelet counts, ×10^9^/L	62.60 ± 28.091	53.20 ± 22.498	0.001
The fifth day platelet counts, ×10^9^/L	96.70 ± 64.839	60.29 ± 29.210	0.000
The seventh day platelet counts, ×10^9^/L	148.64 ± 104.207	74.77 ± 43.380	0.000
The increases of the platelet count in the first 7 days	99.21 ± 102.613	2.08 ± 43.877	0.000
The platelet minimum value during treatment, ×10^9^/L	34.82 ± 19.029	37.54 ± 21.513	0.225
The platelet maximum value within the 14 days, ×10^9^/L	256.89 ± 137.789	125.77 ± 74.076	0.000
The platelet recovery rate within 7 days, n (%)	98, 65.8%	33, 18.5%	0.000
Hemorrhagic score (Godeau et al., 2002) at ICU admission, mean ± SD	5.19 ± 4.311	4.61 ± 4.268	0.228
Decline of hemorrhagic score after 7 days, mean ± SD	2.81 ± 2.856	1.16 ± 2.123	0.000
Incidence of hemostasis in patients with bleeding, n (%)	60, 66.7%	38, 37.3%	0.000
Blood product transfusion			
Patients with PLT transfusion, n (%)	83, 55.7%	81, 45.5%	0.066
Patients with RBC transfusion, n (%)	90, 60.4%	112, 62.9%	0.641
Patients with FP transfusion, n (%)	53, 35.6%	83, 46.6%	0.043
Number of PLT transfusion (U), mean ± SD	1.21 ± 1.661	1.27 ± 2.565	0.841
Number of RBC transfusion (U), mean ± SD	3.639 ± 4.630	5.818 ± 6.858	0.009
Number of FP transfusion (ml), mean ± SD	405.880 ± 713.317	537.500 ± 1039.003	0.273

PLT; platelet; RBC; red blood cell; FP; frozen plasma.

*p* value was generated using t-test, t' test, analysis of covariance or Pearson Chi-Square.

### Effects of rhTPO administration on hemorrhage event and blood product transfusions

The incidence or severity of bleeding between the two groups did not differ at the time of inclusion (Table 1). In total, 58.72% of patients experienced bleeding events. After a 7-day hospitalization in ICU, the rhTPO group’s hemorrhagic score decreased more, compared to that of the no-rhTPO group (2.81 ± 2.856 vs. 1.16 ± 2.123, *p* = 0.000). Bleeding events ceased in 66.7% (60 of 90) of the rhTPO treatment group, compared with 37.3% (38 of 102) in the no-rhTPO group on the 7th day (*p* = 0.000, Table 2).

For all patients, the proportion of transfusion of red blood cells, platelets, and plasma was 61.77, 50.15 and 41.59%, respectively. Compared to the no-rhTPO group, the rhTPO group received less red blood cells transfusions during the 28 days (*p* = 0.009, Table 2) and had a less frozen plasma transfusion rate (*p* = 0.043). A higher proportion of patients in the rhTPO group required platelet transfusion (55.7 vs. 45.5%), although the difference was not statistically significant (*p* = 0.066). However, no difference was noted regarding to the amount of platelet transfusion between the two groups.

### Effects of rhTPO administration on organ functions and infection

We then investigated markers of organ functions (liver, kidney, heart) and biomarkers of infection (WBC, PMNs, Procalcitonin and C-reactive protein). Table 3 presents the results in the time point before, and 7 days after, rhTPO administration or the same time point for the no-rhTPO group. After deducting the effect of the initial baseline value for all relevant indicators in the patients, the declines of hypersensitive troponin I and serum creatinine in the rhTPO group were significantly apparent, compared to those in the no-rhTPO group. However, the other laboratory tests presented no statistically significant differences between the two groups after treatment.

**TABLE 3:** Laboratory tests of all enrolled patients.

Parameters	0 d			7 d		
	rhTPO Group	no-rhTPO Group	*p* Value	rhTPO Group	no-rhTPO Group	*p* Value
cTnI (ng/ml)	1.668 ± 5.750	1.606 ± 5.516	0.921	0.238 ± 0.667	0.496 ± 1.144	0.002
ALT (U/L)	172.356 ± 559.491	92.549 ± 276.123	0.114	48.470 ± 120.097	45.910 ± 94.927	0.868
Cr (μmol/L)	250.275 ± 190.554	225.832 ± 202.858	0.265	141.128 ± 105.244	163.165 ± 117.438	0.006
PT (s)	18.055 ± 8.520	17.186 ± 5.236	0.259	15.923 ± 17.008	17.562 ± 14.106	0.277
FIB (g/L)	3.276 ± 1.835	3.415 ± 2.036	0.521	2.860 ± 1.183	3.360 ± 5.230	0.307
PCT (ng/ml)	25.036 ± 41.973	22.093 ± 46.694	0.553	4.476 ± 25.548	9.880 ± 29.022	0.068
CRP (mg/L)	154.091 ± 97.145	131.129 ± 95.057	0.032	152.140 ± 1088.764	88.902 ± 73.683	0.406
**WBC (×10** ^**9**^ **/L)**	24.254 ± 7.965	23.027 ± 7.838	0.163	13.687 ± 2.867	14.490 ± 3.930	0.124
**PMNs (×10** ^**9**^ **/L)**	20.084 ± 6.837	21.181 ± 6.957	0.153	11.570 ± 2.423	12.249 ± 3.323	0.130

cTnI: cardiac troponin I; ALT: glutamic-pyruvic transaminase; Cr: serum creatinine.

PT: prothrombin time; FIB: fibrinogen; PCT: procalcitonin; CRP: C reactive protein.

WBC: white blood cell; PMNS: polymorphonuclear neutrophils.

*p* value was generated using t-test, t' test or analysis of covariance.

### Effects of rhTPO administration on Platelet recovery rate within seven days

Finally, 11 variables related to platelet recovery rate were analyzed using logistic regression (Table 4). Six variables including “rhTPO administration” were considered independent indicators, and they affected the platelet recovery rate within 7 days (other variables included gender, age, pre-treatment PC, the minimum PC and PSI). Notably, the number of platelet transfusion within 7 days did not influence the platelet recovery rate independently (*p* = 0.064, Table 4).

**TABLE 4:** Multivariate analysis of platelet recovery rate within 7 days.

Variable	Unadjusted			Adjusted		
7-d Platelet Recovery Rate	OR/RR	95%CI	*p* Value	OR/RR	95%CI	*p* Value
Treated with rhTPO	7.545	4.592–12.399	0.000	15.115	7.242–31.549	0.000
Male	0.589	0.359–0.968	0.037	0.301	0.148–0.610	0.001
Age^a^	0.961	0.834–1.109	0.588	0.718	0.574–0.899	0.004
Medical	1.014	0.637–1.615	0.953	1.181	0.580–2.404	0.646
CAP	1.039	0.649–1.666	0.872	1.184	0.574–2.441	0.648
minPC^a^	0.424	0.304–0.590	0.000	0.182	0.095–0.349	0.000
PC1^a^	1.376	0.974–1.945	0.070	2.230	1.185–4.198	0.013
Number of platelet transfusion within 7days	0.681	0.568–0.816	0.000	0.784	0.606–1.014	0.064
APACHE II^a^	0.986	0.812–1.197	0.885	0.920	0.667–1.270	0.613
SOFA	0.913	0.848–0.982	0.014	0.900	0.785–1.031	0.128
PSI^a^	1.196	0.797–1.795	0.387	2.416	1.226–4.759	0.011

a Age: the recoded age; minPC: the recoded minimum platelet counts of patients. PC1: the recoded pre-treatment platelet counts of the patients; PSI: the recoded Pneumonia Severity Index. medical was as opposed to surgical; CAP (community-acquired pneumonia) was as opposed to HAP/VAP (hospital-acquired pneumonia or ventilator-associated pneumonia). APACHE II: the recoded APACHE II, score. OR: odds ratio; RR: relative risk; Adjusted OR, was adjusted by other independent variables; CI: confidence interval. *p* value was generated using logistic regression. The specific methods of recoding are showed in Supplementary Table S1.

Compared to the patients in the no-rhTPO group, those in the rhTPO group had a higher platelet recovery rate (odds ratio, 15.115 (7.242–31.549), *p* = 0.000) 7 days after rhTPO treatment. After 40 years of age, the platelet recovery rate increased by 0.718 times for every 10 years (odds ratio, 0.718 (0.574–0.899), *p* = 0.004). The results before and after adjustment by other covariables are presented in Table 4.

## Discussion

In this study, we found that rhTPO was associated with the increased platelet counts in pneumonia patients with thrombocytopenia. Moreover, we found that rhTPO administration is associated with reduced risk of bleeding and fewer transfusions of RBC and fresh plasma. To the best of our knowledge, it is the first study that explores the benefit of rhTPO on pneumonia patients with thrombocytopenia.

Platelets have long been solely considered as the drivers of hemostasis and coagulation. However, in recent years, multiple biological functions have been discovered in the field of inflammation and immunology, apart from blood clotting (Guo and Rondina 2019; Shannon 2021). The lung is also a primary site for platelet biogenesis and reservoirs (Lefrançais and Looney, 2019), which are responsible for approximately 50% of total platelet production (Lefrançais et al., 2017). Platelets also contribute to the pathophysiology of a variety of lung disorders and to systemic syndromes that involve the lungs (Weyrich and Zimmerman 2013); they have been found to trap, sequester and, in some cases, eliminate invasive pathogens in pneumonia such as pneumococcus, Haemophilus influenzae, Staphylococcus aureus, and viruses (Feldman and Anderson 2020). Thus, platelets play a significant role in restraining bacterial infections to the lung (Amison et al., 2018).

Thrombocytopenia is a recognized severity criteria and a predictor of mortality for hospitalized patients with community-acquired pneumonia (CAP) (Brogly et al., 2007); it is included in the minor criteria for severe CAP defined by the current guidelines to predict ICU admission (Metlay et al., 2019). It is also relevant to poorer in-hospital outcomes and strongly predicts increased long-term mortality due to CAP (Gorelik et al., 2017). In the current study, all patients with pneumonia and thrombocytopenia had very high disease severity scores, such as APACHE II, SOFA, and PSI, suggesting a very critical condition and poor prognosis. In addition, these patients with pneumonia and thrombocytopenia are more likely to develop bleeding, anemia, or receive blood transfusion during hospitalization (Gorelik et al., 2017). Compared with patients without thrombocytopenia, the risk of bleeding is more than three times as high in patients with a low platelet count (Strauss et al., 2002), with bleeding rate ranging from 33 to 52.6% (Vanderschueren et al., 2000; Strauss et al., 2002). In our findings, approximately 60% of the patients with pneumonia and thrombocytopenia experienced bleeding events and received red blood cells transfusion, and about half of them required platelet transfusion.

Previous studies displayed that sepsis patients with prolonged thrombocytopenia and without a relative increase in platelet counts have longer ICU and hospital stays and higher 28-days mortality rates (Akca et al., 2002; Venkata et al., 2013). Further, the daily change in platelet counts is as good as the APACHE II score in predicting mortality, and the blunted rise in platelet counts in critically ill patients is associated with worse outcomes (Nijsten et al., 2000). Therefore, the treatment aimed at rapidly correcting thrombocytopenia perhaps could improve the outcomes of infected patients. Our findings suggested that rhTPO might rapidly increase the platelet counts. The rhTPO group had a lower platelet counts; however, after three, five, and 7 days of treatment, the increase in platelet counts and the growth rate in the rhTPO group were higher, compared with those in the no-rhTPO group. Moreover, more patients who were treated with rhTPO reached clinical recovery of the platelet counts within 7 days.

After the cloning of TPO in 1994, two recombinant thrombopoietic growth factors, namely PEG-reHuMGDF and rhTPO, have been studied in humans in a variety of clinical settings. Development of neutralizing antibodies to endogenous TPO after administration of PEGreHuMGDF is a great concern. rhTPO could also induce the production of antibodies. However, the antibodies are temporary and not neutralizing in nature (Haznedaroglu et al., 2002; Lin et al., 2015). As a humoral growth factor, rhTPO is originally identified by its ability to promote the differentiation of bone marrow hematopoietic stem cells into megakaryocytes and stimulate the growth, proliferation, differentiation and maturation of megakaryocytes (Kuter 2009). rhTPO may act on both the bone marrow and lungs, which are reservoirs for megakaryocytes (Lefrançais and Looney, 2019), to promote platelet production and release into the blood stream. Of note, rhTPO is recommended in multiple guideliens in infected patients with thrombocytopenia (Committee, 2019; Critical Care Medicine Committee of Chinese PLA et al., 2020).

Owing to rhTPO’s remarkable efficacy in elevating platelet counts, resulting in faster decline of hemorrhagic scores and earlier cessation of bleeding events, it can potentially reduce blood transfusion. The rhTPO group seemed to need less red blood cells transfusions during the 28 days. However, the platelet transfusion rate was higher in the rhTPO group. Moreover, this study failed to demonstrate an efficacy of rhTPO in the amount of platelet transfusion and frozen plasma transfusion, which was similar to a report of infection-associated thrombocytopenia (Lin et al., 2015). This lack of difference in platelet and frozen plasma transfusion between the two groups and higher platelet transfusion rate in the rhTPO group are most likely related to some of the following factors. The patients in the rhTPO group were more severely ill and had a lower platelet counts on admission; thus, the doctors in charge were more aggressive in giving platelet and plasma transfusions. In addition, our results showed that the platelet count in the rhTPO group was significantly higher than that in the no-rhTPO group from the third day. However, for the first 2 days, the rhTPO group may need platelet transfusion more.

Moreover, we also found that six variables (treated with rhTPO, gender, age, pre-treatment platelet counts, the minimum platelet counts, and PSI) were independent indicators that affected the platelet recovery rate within 7 days. As platelet transfusions may be a confounding variable for the efficacy of rhTPO, logistic regression was also performed using platelet transfusion as the covariance. However, the results showed that the amount of platelet transfusion within 7 days did not influence the platelet recovery rate independently. The multivariable regression analysis suggested that rhTPO treatment might improve platelet counts and the platelet recovery rate quickly and effectively within 7 days, independent of other treatment.

rhTPO was well tolerated by pregnant patients and sepsis patients, which suggested that rhTPO is a safe drug. In a prospective study, no thrombotic events were observed in any patients through day 15 after sepsis onset (Wu et al., 2014). In addition, rhTPO therapy for patients with ITP during pregnancy with a median follow-up of 53 weeks did not increase thrombosis (Kong et al., 2017). In these two studies, only mild adverse events were observed, including dizziness, fatigue, and pain at injection site. No severe adverse events were reported during the observation period, and no studies were withdrawn because of related adverse event.

Our study has some limitations. First, it was a single-center retrospective study, and the patients in the two groups were not randomized. The generalizability of our conclusion to other populations is not known. Second, baseline platelet counts and disease severity were inconsistent between the two groups when patients were enrolled. Patients in the rhTPO group were enrolled with lower platelet counts and more severe disease. Third, the severity of thrombocytopenia was not graded against the severity of pneumonia. However, patients with pneumonia complicated with thrombocytopenia, whether mild or severe, have higher mortality and higher incidence of bleeding than patients with normal platelet counts (Strauss et al., 2002). Fourth, a well-organized, random, and double-blind clinical trial as well as a dynamically and systematically analysis of the role of rhTPO in pneumonia are needed in future to confirm our phenomenon.

## Conclusion

The study finds that rhTPO administration is related to increased platelet counts, alleviated bleeding, and reduced blood transfusion. Thus, rhTPO may benefit pneumonia patients with thrombocytopenia. Further, rhTPO treatment could be a significant independent indicator that affects the platelet recovery rate. For patients with pneumonia and thrombocytopenia, rhTPO may be an effective therapeutic drug.

## Data Availability

The original contributions presented in the study are included in the article/Supplementary Material further inquiries can be directed to the corresponding authors.
