# Supplementary material for: Administration of recombinant human thrombopoietin is associated with alleviated thrombocytopenia in adult intensive care unit patients with pneumonia: A single-center retrospective study
Source: Front Pharmacol. 2022 Oct 10;13:1007719. doi: 10.3389/fphar.2022.1007719 (PMC9589100; doi:10.3389/fphar.2022.1007719)
Supplement: Supplementary file 1 [file DataSheet1.pdf]

Supplementary Table 1. Methods for recoding independent variables

	Variables	Score
**Age(years)**	<40	1
	40-49	2
	50-59	3
	60-69	4
	70-79	5
	≥80	6
**Platelet count**	51 - 100 $\times10^9$/L	1
	21 - 50 $\times10^9$/L	2
	≤20 $\times10^9$/L	3
**APACHE II**	<15	1
	15-19	2
	20-24	3
	25-29	4
	≥30	5
**PSI**	≤90	1
	91-130	2
	≥131	3

APACHE Ⅱ, Acute Physiology and Chronic Health Evaluation Ⅱ; PSI, the Pneumonia Severity Index

Supplementary Table 2. the use of antimicrobial agents

Antibiotics	**rhTPO group**	**no-rhTPO group**	**P value**
Number, n	149	178	—
Ceftazidime, n (%)	5, 3.4%	6, 3.4%	0.994
Ceftazidime/avibactam, n (%)	3, 2.0%	6, 3.4%	0.517
Cefoperazone/sulbactam, n (%)	82,55.0%	100,56.2%	0.835
Piperacillin tazobactam, n (%)	36, 24.6%	45, 25.3%	0.815
Imipenem cilastatin, n (%)	15, 10.1%	19, 10.7%	0.858
Meropenem, n (%)	8, 5.4%	10, 5.6%	0.922
Vancomycin, n (%)	9, 6.0%	11,6.2%	0.958
Linezolid, n (%)	7, 4.7%	8, 4.5%	0.930

P value was generated using Pearson Chi-Square or the Fisher exact method.
